# Accidental intra-arterial injection: an under-reported preventable never event

**DOI:** 10.1186/cc14246

**Published:** 2015-03-16

**Authors:** M Mariyaselvam, A Hutton, P Young

**Affiliations:** 1 Queen Elizabeth Hospital, King's Lynn, UK

## Introduction

Depending upon the medication administered, accidental administration of medication into the arterial line can cause devastating complications. This wrong-route injection is a never event in the UK but may be under-reported especially when occurring in the unconscious patient who may not notice associated pain temporally. Under-reporting may occur because resultant complications may be delayed a number of hours and the accountable healthcare worker may not recognise or choose not to report the error. In 2008 the UK National Patient Safety Agency (NPSA) reported only 76 incidents related to poor sampling technique but few wrong route arterial injections. Of these 21% suffered moderate to severe harm [1]. The NPSA suggests that training and the use of clear labelling alongside red arterial tubing and standard red lock caps be used to prevent arterial sampling errors.

## Methods

In 2014, we conducted a national postal survey of ICUs in the UK to attempt to determine the rate of accidental intra-arterial injections. The survey was sent to the clinical director of every ICU and they were asked whether they were aware of any unintentional arterial line injection having occurred in their hospital in the last 5 years.

## Results

Of the 56 ICUs that responded, 16 (28.5%) reported that they had personally seen an accidental injection into the arterial line.

## Conclusion

Despite the arterial line safety recommendations made by the NPSA in 2008, we demonstrate that intra-arterial injection is still a problem and that it remains under-reported. Our incidence is likely to be an underestimate as it relies on the recollections of a single individual in each institution. Medical errors can be mitigated by consideration of human factors and system engineering to improve patient safety. A focus on clinical awareness, colour coding and training may lead to improvements; however, institutions and clinical directors also bear a responsibility to prevent never events and a number of engineered solutions are now available such as needle-free non-injectable arterial sampling devices to protect the healthcare environment and make this error impossible [2,3].

## Acknowledgements

Funding from Eastern Academic Health Science Network, UK.

## References

[B1] Rapid response alert 06 NPSA 2008

[B2] Mariyaselvam Anaesthesia 2014 70 51 525308107

[B3] Mukhopadhyay Critical Care 2010 14 R7 10.1186/cc8859 20105285 PMC2875519

